# Fresh Chokeberry (*Aronia melanocarpa)* Fruits as Valuable Additive in Extruded Snack Pellets: Selected Nutritional and Physiochemical Properties

**DOI:** 10.3390/plants12183276

**Published:** 2023-09-15

**Authors:** Agnieszka Wójtowicz, Maciej Combrzyński, Beata Biernacka, Renata Różyło, Maciej Bąkowski, Karolina Wojtunik-Kulesza, Jarosław Mołdoch, Iwona Kowalska

**Affiliations:** 1 Department of Thermal Technology and Food Process Engineering, University of Life Sciences in Lublin, 20-612 Lublin, Poland; agnieszka.wojtowicz@up.lublin.pl (A.W.); beata.biernacka@up.lublin.pl (B.B.); 2 Department of Food Engineering and Machines, University of Life Sciences in Lublin, Głęboka 28, 20-612 Lublin, Poland; renata.rozylo@up.lublin.pl; 3 Institute of Animal Nutrition and Bromatology, University of Life Sciences in Lublin, Akademicka 13, 20-950 Lublin, Poland; maciej.bakowski@up.lublin.pl; 4 Department of Inorganic Chemistry, Medical University of Lublin, Chodźki 4a, 20-093 Lublin, Poland; 5 Department of Biochemistry and Crop Quality, Institute of Soil Science and Plant Cultivation, State Research Institute, 24-100 Puławy, Poland; jmoldoch@iung.pulawy.pl (J.M.); ikowalska@iung.pulawy.pl (I.K.)

**Keywords:** extrusion-cooking, fresh chokeberry, snack pellets, processing conditions, antioxidant potential, physiochemical properties, color profile

## Abstract

In this paper, the nutritional value and (selected) physiochemical properties of extruded snack pellets enriched with fresh chokeberry (*Aronia melanocarpa*) fruits were analyzed from the perspective of being a new product for the functional food sector. The purpose of this study was to determine the effect of the addition of fresh chokeberry and variation in content and screw speed on extruded snack pellet basic compositions, fatty acid profiles, antioxidant activity, as well as water absorption and solubility indexes, fat absorption and color profiles. The obtained results revealed a significant increase in antioxidant activity for all samples (above 90% of free radical scavenging) in comparison to potato-based control samples (just over 20% of free radical scavenging). The total phenolic content assay revealed the most valuable results for samples enriched with 30% chokeberry, while Ultra Performance Liquid Chromatography (UPLC) analysis allowed the determination of the most important phenolic acids. Of interest, chokeberry addition decreased the fat absorption index (FAI) after expansion by frying. Moreover, the highest values of crude protein and crude ash were observed in snack pellets supplemented by the application of 30% chokeberry. In such samples, the crude protein content was at the level of 4.75–4.87 g 100 g^−1^ and crude ash content at 4.88–5.07 g 100 g^−1^. Moreover, saturated fatty acids (SFA) content was lower in snack pellets with chokeberry addition, and increasing the amount of chokeberry additive from 10% to 30% in extruded snack pellet recipes resulted in more than double an increase in polyunsaturated fatty acids (PUFA) proportion in the total fatty acids.

## 1. Introduction

Epidemiological studies have highlighted the link between a diet high in fruits and vegetables and a lower incidence of inflammatory-related disorders [1,2,3]. Fruits are rich in essential nutrients such as vitamins and minerals, fiber and antioxidants, and it is possible to prevent diseases such as diabetes, obesity, heart disease, and even some types of cancer by eating them in the right amounts [4]. Thus, the addition of fruits into highly processed foods may be valuable to increase the level of active substances and enhance the nutritive content of a commonly over-consumed food item.

Many bioactive compounds with a wide range of health-promoting properties can be found in black chokeberry (*Aronia melanocarpa*) [5,6,7]. Indeed, it has been found that black chokeberry has 10 times the antioxidant capacity of other berries [8], outranking currants, cranberries, blueberries, elderberries and gooseberries. Moreover, it has one of the greatest quantities of sorbitol. These fruits are also abundant in polyphenolics and thus have one of the highest levels of antioxidant and anti-inflammatory activity among all berry fruits [9]. Beyond the aforementioned, chokeberry bioactive components have anti-cancer, anti-bacterial and anti-diabetic activities, as well as hepatoprotective, cardioprotective and neuroprotective potential [10].

The polyphenolic compounds in its fruits include flavonoids, phenolic acids, proanthocyanidins [11] and hydroxycinnamic acids. It has been observed that proanthocyanidins are the main factor influencing the antioxidant activity of chokeberry, while proanthocyanidins are the most powerful antimicrobial agents in this fruit [12]. Kulling et al. [13] found the concentrations of 48 compounds identified as volatile constituents of black chokeberry. Herein, the main compounds were benzaldehyde cyanohydrin, hydrocyanic acid and benzaldehyde, as well as a series of benzene derivatives, including benzyl alcohol, 2-phenylethanol, phenylacetaldehyde, salicylaldehyde, acetophenone, 2-hydroxyacetophenone, 4-methoxyacetophenone, phenol, 2-methoxyphenol and methyl benzoate.

Promising therapeutic compounds extracted from the Aronia fruit include cyanidin-3-O-galactoside, chlorogenic acid, quercetin and ursolic acid. These compounds are being tested in clinical trials as cancer treatments [14]. Chokeberries are also a potentially rich source of minerals such as K, Ca, P, Mg, Na, Fe and Zn [15]. According to recent research, the element with the highest content in chokeberry fruits is potassium (approximately 4000 mg kg^−1^), followed by calcium (about 1200 mg kg^−1^) and magnesium (about 500 mg kg^−1^). Consumption of black chokeberry fruit and products based on it can improve overall health due to the beneficial effects of these macronutrients on the cardiovascular, motor and immunological systems [16]. Summing up the health benefits of chokeberry, it should be emphasized that the pharmacological actions of these fruits can help treat a variety of human disorders, including cardiovascular disease, hyperlipidemia, hypercholesterolemia, hypertension and diabetes. Furthermore, black chokeberry has also been used to treat diseases such as leukemia, breast cancer, and intestinal cancer, as well as cancer stem cells. It is clear that the nutritional value and efficacy of black chokeberry have piqued the interest of medical professionals [17].

The above-mentioned findings could be important in future research on chokeberry-based functional food products [18]. Chokeberries are rarely directly consumed because of their tart flavor, but they are used in the food industry to make juices, nectars, syrups, jams, preserves, wines, tinctures, and fruit desserts [5,19]. The antioxidant potential of chokeberry fruit, however, may change during processing. Therefore, it is necessary to determine how processing affects these properties [20,21].

Currently, extrusion-cooking processes are increasingly used in snack food processing [22,23]. Controlling the effect of the extrusion-cooking process on the changes in functional components, as well as physical properties and texture is still one of the biggest problems with its development [24,25]. Still, the inclusion of health-promoting components can improve the nutritional potential of this type of product [26,27,28], and chokeberry is a potential candidate for enhancing this.

In order to produce extruded snacks or other products with high nutritional quality and acceptable sensory profile, it is crucial to understand the physical and chemical changes that occur during extrusion-cooking [29,30]. Therefore, it is necessary to conduct a series of studies to determine what qualitative changes are caused by this process. Research conducted so far in the field of extrusion-cooking has shown that the health benefits of chokeberry consumption can be accessed through properly designed directly expanded functional snacks and breakfast cereals [31]. Indeed, the retention of functional compounds from chokeberry (procyanidins or hydroxycinnamic acids), was not affected by changes in processing conditions, as reported by Schmid et al. [32] in adding 25% of chokeberry pomace to textured cereals, or by Oniszczuk et al. [33] if 5, 10, 15 and 20% of dried chokeberry fruit was used in extruded instant porridge based on corn grits.

Another study examined the effect of the extrusion process on the physical properties and nutritional composition of black chokeberry pomaces. It was discovered that the extrusion-cooking process reduced the content of anthocyanins and fiber while increasing the contribution of simple sugars [2]. It was also found that extruded corn puffs with chokeberry pomace added (5–20%) had a much higher antioxidant activity than the experiment’s control [34]. However, other researchers concluded that the addition of dried chokeberry fruit to gluten-free directly expanded corn snacks should not exceed 15% because the larger addition resulted in an unfavorable increase in product hardness [35]. In still other studies, extrudates containing 25% chokeberry pomace powder were found to offer acceptable techno-functionality and sensory physical properties, while higher fruit pomace addition resulted in reduced expansion and cell pore size of slightly darker and softer extrudates [4]. The application of fresh chokeberry pulp in the composition of snack pellets has not been investigated yet, hence, beyond the scientific, the practical aspects of the development and processing of new types of functional products are also advantages of the presented research.

The aim of this study was to determine the effect of the addition of fresh chokeberry and processing variables on extruded snack pellet’s basic composition, fatty acid profile, antioxidant activity, selected physical properties and color profiles.

## 2. Results and Discussion

### 2.1. Results of Chemical Composition of Snack Pellets

In our work, we determined the proximate composition of extruded snack pellets supplemented with various chokeberry addition, as well as their fatty acid profiles, and saw that the proximate composition and profile of fatty acids of the produced snack pellets differed significantly. The amount of fresh chokeberry applied in the recipes of snack pellets was the main factor influencing such differences (Table 1).

Application of 30% fresh chokeberry to the extruded recipes produced improvement of the basic chemical components content in relation to samples with the addition of 10% of chokeberry and to control samples if screw speeds of 60 and 100 rpm were applied. We found an increase in crude ash and thus associated enhanced micro- and macroelements content, crude fat and total protein content and eligible dietary component in the derived snack products. Thus, the increase in the total protein content in snack pellets supplemented with the addition of the highest chokeberry amount enhanced the nutritional value of the supplemented snack pellets. In the control snack pellets (without chokeberry additives), the protein content was the lowest due to the low amount of proteins in basic potato components of such recipes. In control samples, the protein content was between 3.46–3.64 g 100 g^−1^, the content of crude ash was between 3.70–3.76 g 100 g^−1^, and crude fat was between 0.07–0.11 g 100 g^−1^. The highest values of these components were observed in snack pellets supplemented by the application of 30% of chokeberry. In such supplemented samples, the crude protein content was between 4.75–4.87 g 100 g^−1^, crude ash content between 4.88–5.07 g 100 g^−1^, and crude fat between 0.15–0.21 g 100 g^−1^ (Table 1). The lower-than-expected crude fiber content in tested extrudates might be an effect of the macromolecular compounds’ degradation during the extrusion-cooking process and the formation of some complexes between components or destruction of fibrous components under the high temperature and pressure treatment of processed materials [36,37]. The basic chemical components were strongly correlated with the amount of fresh chokeberry addition. The increase in fresh fruit in the recipe affected crude ash content (r = 0.991), crude protein (r = 0.992), crude fat (r = 0.750) and total carbohydrates content (r = −0.963). We also noted a strong correlation between ash and protein content (r = 0.979), as well as crude fat and ash (r = 0.715) and protein (r = 0.750). When the amount of ash, protein and fat increased, the total carbohydrate level in snack pellets decreased significantly (r = −0.952, −0.958, −0.716, respectively).

The effect of chokeberry addition and processing variables on fatty acids composition in snack pellets is presented in Table 2. Control potato-based samples without additives showed a higher amount of saturated fatty acids as compared to fortified samples, especially C 16:0. Content of C 18:1 n-9 acids were the highest if 10% of fresh chokeberry was added. We noted in samples fortified with 30% fruit, the highest content of C 18:2 n-6 and C 18:3 n-3 acids. Furthermore, the saturated fatty acids (SFA) sum was lower in snack pellets with chokeberry addition and the SFA content fell with the increased level of additive, the lowest content being reached when 30% additive was applied under processing conditions at 36% moisture level and 100 rpm. Minor differences in fatty acids composition in comparison to control were indicated if only 10% of fresh chokeberry was used in the recipe, but increasing the amount of chokeberry additive from 10 to 30% as an enriching component in extruded snack pellet recipes brought about a more than double increase in PUFA proportion in the total fatty acids amount, and positively, a simultaneous decrease in SFA proportion in total fatty acids amount was noted. This may enhance the nutritional value of chokeberry-supplemented snack pellets [38]. We found that fatty acids composition was significantly correlated with the content of fresh chokeberry addition, including the effect on SFA (r = −0.934), MUFA (r = −0.857) and PUFA (r = 0.936). We also found strong negative correlations between fatty acids and basic components in the tested snack pellets enriched with fresh chokeberry. Here, SFA was negatively correlated with crude ash (r = −0.924), crude protein (r = −0938) and crude fat (r = −0726), and positively with carbohydrates (r = 0.909). Our work also revealed slightly lower negative correlation coefficients between MUFA and ash (r = −891), protein (r = −0.815), carbohydrates (r = 0.809) and SFA content (r = 0.782). In contrast, we obtained strong positive correlations between PUFA and ash (r = 0.954), protein (r = 0.911) and fat content (r = 0.747). Moreover, we noted positive relations between PUFA and carbohydrates (r = −895), SFA (r = −0.915) and MUFA (r = −0.967).

Bajramova and Spégel [39] indicated nineteen different fatty acids in fruits such as goji berry, white mulberry and cranberry (known as “superfruits”) and banana, apple, and strawberry. Concentrations of fatty acids (FA) between 0.018 and 9.40 mg per g of dry sample were detected, and the ratio of unsaturated to saturated FAs was the highest in strawberries and goji berries. They concluded that the fatty acids profile in the tested fruits suggested that apples and cranberries demonstrate the most beneficial lipid profile to the human body [39].

### 2.2. Antioxidant Activity, Content of Polyphenols and Phenolic Acids of Snack Pellets

Chokeberry is a plant rich in antioxidants and other valuable pro-health compounds [40,41]. Among these are polyphenols. Their consumption counteracts oxidative stress, and is considered pro-health. According to published research, chokeberry fruits had a total polyphenol content ranging from 1022 mg in 100 g of fresh weight (FW) to 1795 mg in 100 g FW, and their oxygen radical absorbance capacity (ORAC) antioxidant activity ranges from 109 to 191 mol TE g^−1^ FW [42]. The highest ORAC and TRAP (total radical trapping antioxidant potential) antioxidant activity was found in the secondary phenolic components, quercetin and epicatechin. Chokeberry fruits are often used in the food industry as additives or alone in raw or processed form (dried, lyophilized, as extracts). One possible way to consume these valuable fruits is as functional food enriched with this additive as it gains a high content of antioxidants. Still, some loss of the phenolic compounds has been observed during processing, especially through extrusion, for example, due to the thermal, oxidative or enzymatic degradation where several factors such as pH, processing temperature or presence of enzymes may affect the content of thermolabile plant compounds [19,34]. Previous research has found that textured cereal extrudates containing 25% chokeberry pomace, however, demonstrate acceptable physical properties, with anthocyanins degraded to about 70% and phenolic acids and flavonols fully retained [32]. In addition, the retention of procyanidins and hydroxycinnamic acids was not affected by changes in screw speed (300, 500 rpm) and barrel temperature (100 and 140 °C) according to research on high-temperature, short-term extrusion-cooking processing of corn snacks with the addition of chokeberry extract [31].

**Figure 1:**
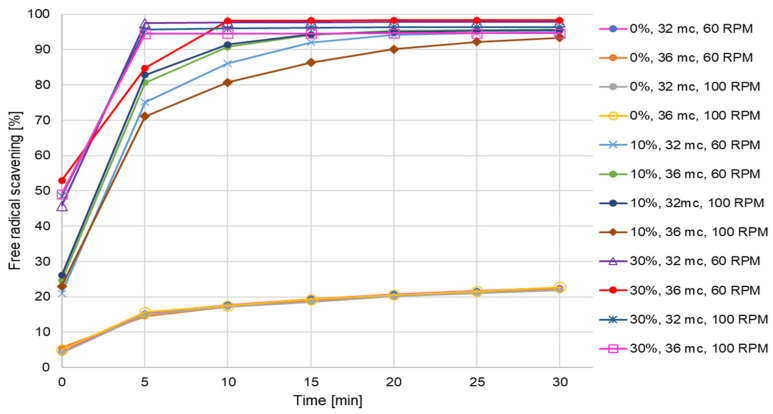
Free radical scavenging activity of selected extract samples obtained with use of DPPH method (%—additive level; mc—moisture content; RPM—screw speed).

DPPH (2,2-Diphenyl-1-picrylhydrazyl) assay allows determination of the free radical scavenging ability of functional food products enriched with chokeberry (10% and 30%) as compared to control potato-based samples. In undertaking this work, we noted the valuable influence of the additive on antioxidant activity. Indeed, we observed a significant increase in the activity of the samples in comparison to the basic potato recipe, namely, 73% and 76% enhancement for 10% and 30% addition of the fruit, respectively (Figure 1).

What we found to be equally important was the speed of reaction. In the case of samples enriched with the fruits, high activity was observed immediately after the onset of the reaction, and the extracts scavenged above 80% of free radicals within 5 min. The feature is significant for future consideration of the antioxidant activity of studied samples. The study results based on both the antioxidant method (DPPH), as well as analysis of polyphenols content (Folin–Ciocalteu, UPLC chromatography) confirmed the high pro-health activity of the functional food product. Taking into account the obtained results, significant growth of antioxidant activity was apparent even with a small addition (10%) of fresh chokeberry. Processing variables, namely, moisture level and screw rotation speed (rpm) are parameters having a potential influence on the pro-health properties of the studied food products. In terms of the obtained results, however, we found no significant influence of the factors. In each case, the analyzed extracts reached activity above 90% (Figure 1). Nevertheless, the highest free radical scavenging was observed if the initial moisture of components was at 36% and when processed at 60 rpm for both contents of fresh chokeberry in the recipe.

Trolox equivalent antioxidant activity (TEAC) and total phenolic content (TPC), which, as active antioxidants, can be considered responsible for the high antioxidant activity of the analyzed samples. Considering the total phenolic content, the most valuable results were obtained for samples enriched with 30% of chokeberry and this simultaneously impacted upon higher antioxidant activity. The outcome of the studies explicitly indicated the valuable improvement of pharmacological properties as being result of chokeberry addition. Both antioxidant activity, as well as end phenolic compound content, demonstrated significant improvement resulting from the increase in the fresh chokeberry addition (Table 3). We also noticed that the pro-health properties increased with the content of the additive and the enhancement was significant, with r = 0.991 for TPC and r = 0.787 for TEAC. Moreover, TPC was strongly positively correlated with the content of basic chemicals such as ash (r = 0.992), protein (r = 0.973), fat (r = 0.751) and MUFA (r = 0.959), but negatively correlated with fatty acids (r = −0.911 and −0.906 for SFA and PUFA, respectively) as well as carbohydrates content (r = −0.943).

There is an explicit dependency between the content of chokeberry and the content of polyphenols (Table 3). Nevertheless, even 10% of the fruit addition positively influenced the content of active compounds, which resulted in a significant increase in free radical scavenging activity in comparison to potato-based control samples.

TEAC value obtained for the most active sample with 10% additive is equal to 160.00 µg g^−1^, whereas the value for the most active sample with 30% additive is equal to 163.67 µg g^−1^ (Table 3). Hence, the sample enriched with 30% chokeberry using 32% moisture level and 60 rpm screw speed during production demonstrated the most valuable antioxidant activity. Overall, the lack of changes in the activity indicated the high antioxidant properties of the studied snack pellets. This is important from the point of view of production or taste because even a small (10%) addition of fresh aronia fruit significantly improves the health-promoting properties of food products. Furthermore, the obtained results of total phenolic content and antioxidant activity were significantly correlated (r = 0.710). In addition, antioxidant activity was positively correlated with ash and protein content (r = 0.725 and 0.829, respectively), but negatively with carbohydrates and SFA (r = −0.779 and −0.718, respectively).

Besides the additive content, our work indicated that production parameters have only a slight influence on snack pellet bioactivity. This degree of dependency may be related to the possibility of decomposition of active compounds under the influence of certain production factors. Nevertheless, the extrusion-cooking process may reduce the bioactive substance content and simultaneously enhance the bioavailability of valuable nutritional components in the human body due to more intense absorption of bioactive compounds from the supplemented food products [43,44]. Analysis of the obtained study results did not indicate a negative influence of the used processing parameters. In all cases, the antioxidant activity was high, similar to the content of polyphenols and selected phenolic acids. What is more, in assessing the activity within a group containing the same amount of additive, the activity was very similar. This underlines the lack of negative impact of production parameters on antioxidant activity. The study of Oniszczuk et al. [44] confirms that high-temperature extrusion processing does not deactivate polyphenolic antioxidant compounds, in this case, raw Tilia inflorescence, when extruded instant gruels supplemented with up to 20% of linden flowers were tested. Similar findings were noted by Wójtowicz et al. [45] in corn puffs supplemented with Dracocephalum moldavica leaves up to 20% or in multigrain fried snacks made from pellets supplemented with Moldavian dragonhead seeds up to 22% [46]. These fully demonstrate that high temperature short time (HTST) extrusion-cooking processing is not destructive with regard to the functional components present in plant materials added to enhance the antioxidant potential of snack foods.

In our study, we determined the phenolic acid content of the tested samples via DPPH assay, and the best results that were obtained for functional snack pellet products were: 30% addition of fresh chokeberry at 36% mc, 60 rpm. Here, the following phenolic acids were quantified: protocatechuic—24.44 ± 2.18 μg g^−1^, p-OH-benzoic—0.22 ± 0.04 μg g^−1^, caffeic—153.16 ± 11.23 μg g^−1^, p-coumaric—9.48 ± 0.86 μg g^−1^, ferulic—8.56 ± 0.56 μg g^−1^, sinaptic—3.05 ± 0.12 μg g^−1^ and salicylic—0.40 ± 0.04 μg g^−1^. Vanillic acid and syringic acid were below the limit of quantification (LOQ). The chromatogram obtained for the tested snacks processed at 36 mc and 60 rpm with 30% chokeberry addition is presented in Supplementary Materials as Figure S1.

### 2.3. Content of Anthocyanins

Anthocyanins are flavonoid compounds responsible for the color of fruits and flowers. In addition to aesthetic values, they are extremely important for plant survival and significantly improve pro-health values for people who consume them. There are numerous study results indicating high anthocyanin content in chokeberry fruits. In accordance with Denev et al. [6], the total amount of these in fresh fruits varies from 357 to 1790 mg/g fresh weight. Ultra Performance Liquid Chromatography (UPLC) analysis has revealed the plant metabolites that can be found in snack samples enriched with chokeberry (Table 4).

The results we obtained indicate that cyanidin-3-galactoside was the predominant anthocyanin, whereas cyanidin-3-arabinoside content was also high. In contrast, cyanidin-3-glucoside and cyanidin-3-xyloside were found to be below the limits of observation. Similarly to antioxidant activity, 30% of fruit content displayed the highest anthocyanin content. The most valuable production parameters were 32 mc and 100 rpm and 36 mc and 100 rpm. Our work was found to be in accordance with that of Kasprzak–Drozd et al. [1] and [47].

The health promoting values of anthocyanins were assessed by Wen et al. [41] who determined that the compounds had a positive effect on neurodegeneration. Accordingly, the purified compounds cyanidin 3-O-galactoside and cyanidin 3-O-arabinoside exhibited significant influence on the reduction in oxidative stress and β-amyloid levels, nevertheless, the mechanism of action is still unknown.

The high content of compounds that have not been destroyed during food production speaks for the enrichment of food with chokeberry, which, showing certain health-promoting properties, can be considered a functional food.

### 2.4. Physical Properties of Snack Pellets

Water Absorption Index (WAI) measurement is considered a suitable method for determining the extent of starch gelatinization and dextrinization during food extrusion. The WAI of snack pellets supplemented with chokeberry and processed under various conditions are presented in Table 5. Our research shows that the addition of chokeberry fruit induced a decrease in the water absorption of snack pellets, as compared to the water absorption of ordinary potato snack pellets. A similar tendency was observed by Drożdż et al. [34] for extruded corn snacks with chokeberry press residue. In our work, the WAI values were from 2.79 g g^−1^ for snack pellets with 30% chokeberry addition processed at 100 rpm, to 3.92 g g^−1^ for snack pellets without fruit addition processed at 60 rpm. In general, we can state that the increase in the chokeberry content in the raw material mixture increased the water absorption of the snack pellets. However, in the case of snack pellets obtained with a screw speed of 100 rpm and a mixture moisture content of 34 and 36%, a decrease in the WAI value was observed with an increase in the amount of fruit additive. Overall, our experiment indicates that increased screw speed and increase in initial raw materials moisture level results in increased WAI values.

Water Solubility Index (WSI) is commonly used as suitable marker describing the extent of starch dextrinization and fragmentation during the extrusion-cooking of food products. Results presented in Table 5 show that, in general, increased chokeberry addition in snack pellet recipes brought about lower WSI of snack pellets with a significant correlation (r = 0.893). In contrast, however, for snack pellets processed with 36% moisture content, the WSI values increased with increasing levels of fruit addition.

Our work revealed that the control sample based on potato components processed at 100 rpm with 36% of initial moisture content in the blend (23.40%) displayed the highest WSI. For other snack pellets without fruit addition, WSI results were lower, especially if moisture content or screw speed was low. The WSI values of products supplemented with fruit were from 7.69% for snack pellets with 30% chokeberry addition processed at 60 rpm and 32% of blend moisture, up to 20.82% for pellets supplemented with 10% chokeberry processed at low rpm and high moisture. In general, it can be stated that the increase in screw speed increases the WSI of the snack pellets with chokeberry addition because of more intensive treatment at higher screw rotations that generate higher shear forces during the extrusion-cooking. This high shearing intensity may have a negative impact on processed components making them more fragmented and thus much easier to elute during WAI/WSI testing. The WSI of the tested samples was significantly correlated with some chemical components such as ash and protein (r = 0.863 and 0.887, respectively), MUFA (r = 0.742), TPC (r = 0.873) and TEAC (r = 0.840) and negatively with carbohydrates content (r = −0.841) and sum of SFA (r = −0.793).

Our research results reveal that chokeberry addition significantly decreased the fat absorption index (FAI) after expansion by frying (r = −0.909) (Table 5). This is probably due to the partial replacement of starch with fruit-derived fiber, sugars and pectins. The fat absorption index values were from 3.23% for snack pellets with 30% chokeberry addition processed at 60 rpm and 32% of moisture content, to 40.20% for snack pellets without fruit addition processed at 60 rpm and 36% moisture content. Similar tendencies in FAI changes were observed by Lisiecka et al. [48] in the case of snack pellets with the addition of fresh leek and onion pulps. Moreover, we noted that the FAI was correlated with several features of snack pellets supplemented with fresh chokeberry addition. We saw significant negative correlations between fat absorption and ash (r = −0.882), protein (r = −0.893), MUFA (r = −0.796), TPC (r = −0.887), TEAC (r = −0.799), as well as with WSI (r = −0.840), while positive relations were found with carbohydrates content in snack pellets (r = 0.956), SFA (r = 0.827) and PUFA (r = 0.708).

During the processing of starch-based potato crisps, a starch gel network is created that is sufficiently strong to resist expansion and avoid structural collapse during deep-frying. Reyniers et al. [49], when testing potato crisps (also referred to as indirectly expanded or restructured potato chips) expanded by the frying process concluded that the replacement of water by oil explains the direct relationship between water loss and oil uptake. During frying, the water evaporates from the matrix and the oil is absorbed into the voids formed by the evaporated water. If an overly strong internal structure is formed during processing through the use of additives, dense and hard products with small pores may form, and, hence, oil absorption is reduced. In addition, depending on processing temperature and shear intensity, gelatinization of starch occurs and further gelatinization and fast water evaporation under frying allow pronounced swelling that may affect product texture and lipid content [49]. In related experiments by Lisiecka et al. [50], fresh carrot pulp addition to potato-based recipes reduced the number and pore size of crisps expanded by various applied methods. Lisiecka et al. [50] also noticed that an increased level of fresh beetroot pulp as a supplementary ingredient in snack pellets recipe created a denser deep-fried snack internal structure as compared with that of deep-fried snacks based on potato components with lower fat intake.

### 2.5. Color Profile of Snack Pellets

Table 6 presents the results of the color profile evaluation for the tested snack pellets according to the CIE-Lab scale.

In our work, as compared to control samples based on potato recipes, snack pellets with the addition of chokeberry were found to be much darker due to the intensive purple color of the fruits applied. In contrast, potato-based products showed a lightness ranging from 55.44 to 60.77. Herein, lower screw rpm induced greater snack pellet lightness. When 10% of chokeberry was added, the L* intensity was lower (33.41–35.87) by about 40% compared to the control sample lightness. We found a very intensive lightness decrease in samples supplemented with 30% of chokeberry. In these samples, L* values were 29.14–29.69 and were very similar and not dependent on the processing conditions. However, a significant negative correlation in L* value was evident with an increase in chokeberry content (r = −0.853). Wang et al. [52] reported the effect of higher extrusion temperature on the darkening of extrudates based on protein content. Herein, as processing temperature increased, b* intensity decreased. In our study, the lightness of the tested snack pellets enriched with fresh chokeberry showed significant negative coefficients of correlation with the content of ash and protein (r = −0.800 and −0.886, respectively), TPC and TEAC (r = −0.786 and −0.981, respectively) and WSI (r = −0.862) but we saw positive relation between L* and carbohydrate content and SFA (r = 0.860 and 0.787, respectively, as well as with FAI (r = 0.864).

We also noted low values of red tint (a*) in snack pellets based only on potato components. Starch grits and flakes are white-yellow in color, so only slight differences were noted between the control samples. We did not observe the effect of processing conditions on the a* (redness) of potato-based control samples. However, in the study samples, with higher chokeberry content, the a* values fell due to the formation of more dark-brown colors in the extruded half-products when the additive levels were very high. Changes in the redness of samples with chokeberry addition may be the effect of thermal degradation, Maillard reaction or oxidation of red pigment (anthocyanin) in the extruded samples, as well as due to the presence of reducing sugars (as glucose and fructose in chokeberry) or the hydrolysis of starch or sucrose into reducing sugars through the shearing effect of processing [53,54]. Thus, in the tested samples, the red tint is transferred into a brown shade, and the final snack pellets fortified with fresh chokeberry became more brown than red. No significant correlations were found between a* coordinate and other features of the tested snack pellets.

The yellowness of control potato-based pellets without additives was intensive, and b* value varied from 10.81 to 13.42 in the control samples. The application of even 10% of fresh chokeberry significantly lowered the yellowness of snack pellets. Here, the range of the b* coordinate was between 1.26 to 2.29, with a slight intensive yellow tint observed in samples processed at the highest moisture (36%) and the highest screw speed (100 rpm). This outcome may be due to the shorter and less intensive treatment and lower residence time inside the extruder, as well as the higher moisture of the material. Hence, the lesser effect of shear forces during processing. Increasing the amount of chokeberry to 30% caused further lowering of a yellow tint to 0.64–0.84 (Table 5) with a high correlation coefficient (r = −0.805) and with the negligible applied effect of processing variables. Moreover, we noted several significant correlations between b* values and the snack pellet-tested properties. Accordingly, our work revealed certain negative coefficients between yellowness and ash and protein contents (r = −0.749 and −0.844, respectively), TPC and TEAC (r = −0.731 and −0.986, respectively) and WSI (r = −0.851), as well as some positive correlations between b* and carbohydrates and SFA (r = 0.789 and 0.751, respectively, and FAI (r = 0.786). What is more, b* was also strongly correlated with the lightness of the tested snack pellets (r = 0.985).

All the tested samples fortified with fresh chokeberry addition showed visible color change when compared with potato-based samples (controls), and reached total color change index Δ*E* values from 24.26 to even 32.96 if 30% of fruit was added in the recipe (r = 0.837). As explained by Adekunte et al. [55], Δ*E* differences in perceivable color may be classified as small differences (below 1.5), distinct (1.5–3.0) and very distinct (over 3.0), thus, the presented values are very distinct and easily noticed. The total color difference is considered as one the most sensitive parameters with regard to color degradation during storage or treatment, e.g., in strawberry jam [56], strawberry and blackberry purees [57], tomato juice [55] or carrot and tomato juices [58]. In analyzing correlations of Δ*E* with other tested parameters, we found opposing correlations with similar values of r coefficients such as in the L* and b* color coordinates. This is the effect of the Δ*E* calculation method including all L*, a* and b* values when used to obtain the total color change index.

### 2.6. PCA Analysis

**Figure 2:**
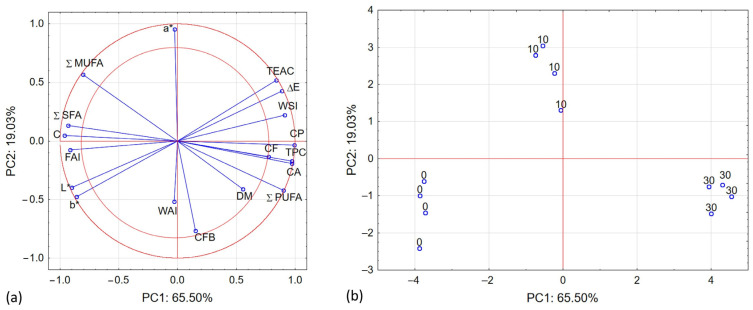
Loading plot (**a**) and score plot (**b**) of the principal component analysis (PC1 and PC2) carried out for addition of chokeberry and tested parameters.

Performing the Principal Component Analysis (PCA) allowed obtaining 11 new variables, of which the first two principal components describe 84.52% of the variability of the system. In Figure 2a, the parameters that occur between the two red circles have the greatest impact on the variability of the tested system (Figure 2a). Accordingly, parameters: L*, b*, a*, Δ*E*, FAI, C, TEAC, WSI, CP, CF, TPC, CA, Σ SFA, Σ MUFA and Σ PUFA have the greatest influence on the variability of the system. CFB and DM have a slightly smaller impact and WAI has a very weak impact. We saw a strong positive correlation between L*, b*, a*, FAI, C, Σ SFA, and we noted a slightly weaker, but also a positive correlation between L*, b*, FAI, C, Σ SFA and Σ MUFA. Moreover, we noted a strong and positive correlation between the parameters TEAC, Δ*E*, WSI, CP, CF, TPC, CA and Σ PUFA. In turn, we saw a strong negative correlation between L*, b*, FAI, C, Σ SFA, Σ MUFA and TEAC, Δ*E*, WSI, CP, CF, TPC, CA Σ PUFA. Overall, our work revealed no correlation between the listed parameters having the greatest impact on the variability of the system and parameter a*.

The PCA analysis shows that the first main component of PC1 in as much as 65.50% describes the case of using the chokeberry supplement (Figure 2b). Positive values of the PC1 main component describe the results for a higher content of chokeberry additive, and negative values of the PC1 main component describe the results for no chokeberry additive. In addition, the second main component (PC2) in 19.03% describes the case of the amount of use of chokeberry additive. Positive PC1 values and negative PC2 values mean a higher content of Aronia addition, and negative PC1 values and positive values of the second PC2 component describe a lower value of chokeberry addition. The PCA analysis for screw speed and moisture level did not show any influence on the variability of the system.

**Figure 3:**
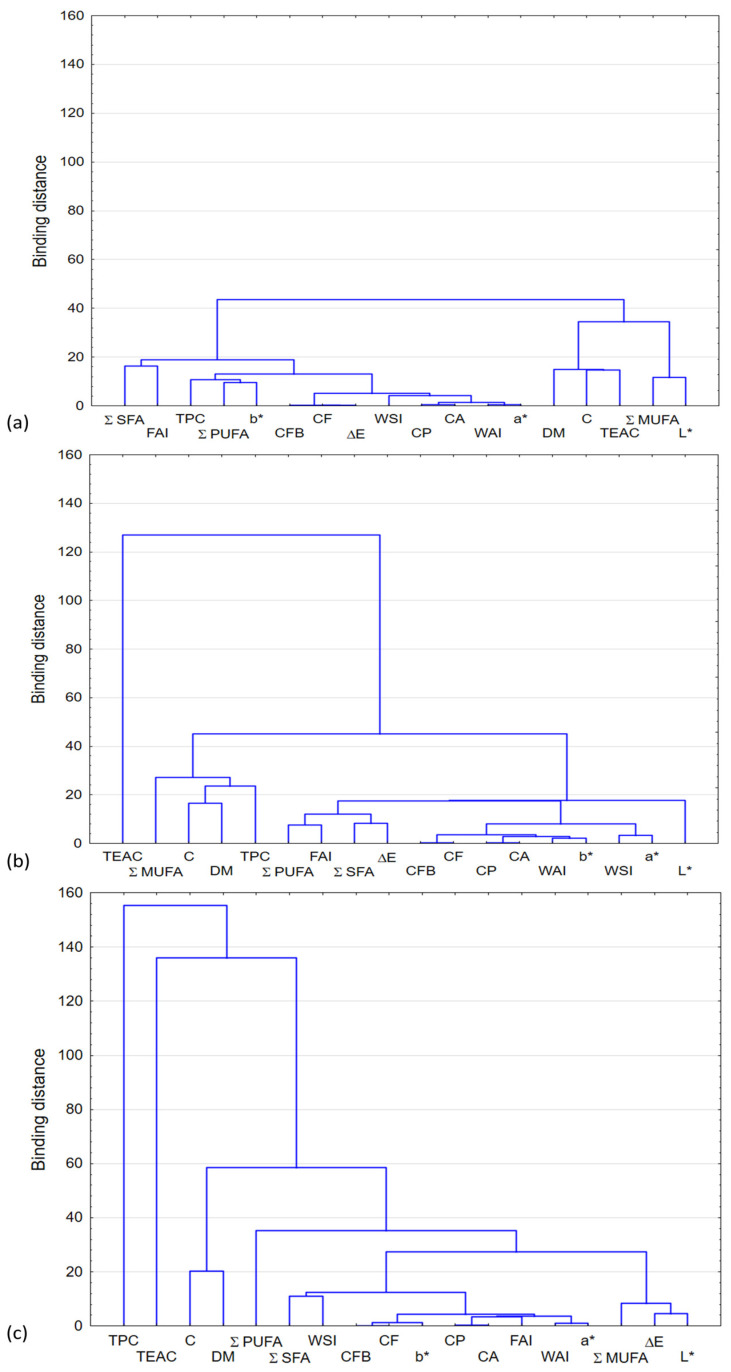
Dendrogram of hierarchical cluster analysis for the snack pellets enriched with fresh chokeberry addition: (**a**) control sample without additive; (**b**) snack pellets with 10% of chokeberry; (**c**) snack pellets with 30% of chokeberry.

Cluster analysis was used for exploratory data analysis (Figure 3), and its aim was to arrange objects into groups in such a way that the degree of linking objects with objects belonging to the same group was as large as possible, and with objects from other groups as small as possible.

Cluster analysis only detects structures in the data without explaining why they occur, and the results of the cluster analysis show that in the absence of chokeberry addition, there are two different groups (Figure 3a). In one, the parameters DM, C, TEAC, Σ MUFA and L* are concentrated. The other parameters are concentrated in the second group. Cluster analysis for two amounts of chokeberry addition does not show a grouping of results (Figure 3b,c).

## 3. Materials and Methods

The evaluation of the manufacturing process and the analysis of the chemical and physical properties of snack pellets with the addition of chokeberry were carried out depending on the additive content and processing conditions.

### 3.1. Raw Materials

The development of new types of extruded snack pellets was carried out on the basis of previously composed basic recipes, which included:

– potato starch SUPERIOR STANDARD (Przedsiębiorstwo Przemysłu Ziemniaczanego Bronisław S.A., Bronisław, Poland); proximate composition (dry weight): moisture 12.87%, protein 0.39%, fat 0.0%, ash 0.34%, fiber 4.94%,
– potato flakes (Zakłady Przemysłu Ziemniaczanego w Lublinie, Lublin, Poland); proximate composition (dry weight): moisture 9.26%, protein 8.22%, fat 0.02%, ash 3.82%, fiber 15.38%,
– potato grits (Zakłady Przemysłu Ziemniaczanego w Lublinie, Lublin, Poland); proximate composition (dry weight): moisture 7.51%, protein 8.27%, fat 0.33%, ash 3.98%, fiber 11.28%,
– vegetable oil (Zakłady Tłuszczowe “Kruszwica”, Kruszwica, Poland),
– sugar purchased at a Lidl store (Lublin, Poland),
– salt purchased at a Lidl store (Lublin, Poland).

At the initial stage, dry mixtures of basic components were prepared as control samples. Organically grown chokeberry (ANREKO Andrzej Gębka, Jakubowice Konińskie, Poland) was applied to develop new recipes of snack pellets in which the percentage of fresh chokeberry was set at 10% and 30% of the total sample mass. The proximate composition of chokeberry was as follows (fresh matter): moisture 69.74%, protein 0.7%, simple sugars 17.6%, phenols total 2%, organic acids 1.3%, fat 0.14%, ash 0.40%, fiber 5.62% (producers’ data). Immediately after receiving chokeberry from the supplier, the fruits were crushed and homogenized utilizing a cup blender Germin MAX-1050-W (Germin, Berlinger, Germany). These were bagged and frozen. The frozen chokeberry was pulled out 12 h prior to the preparation of the mixtures for thawing. We mixed the dry ingredients with the fresh fruit pulp to obtain homogenous compositions. These were passed through a 1 mm sieve. We stored the prepared mixtures at a reduced temperature (approximately 6 °C) to stabilize the moisture content for 24 h. In the next step, we checked the moisture content using a Radwag MA 50 R weighing drier (Radwag, Lublin, Poland) and, depending on the moisture content, the blends were moistened to 32% and 36% moisture by adding the appropriate amount of water and pass through the sieve again.

### 3.2. Extrusion-Cooking of Snack Pellets

We carried out the snack pellet extrusion process using a prototype single-screw extruder EXP-45-32 (Zamak Mercator, Skawina, Poland) with a working screw configuration of L/D = 20 and a screw speed of 60 and 100 rpm. Each mixture placed into the feeding section was extruded at the temperature 40–80–95–75 °C and the dough was forced through the extruder die with a single flat opening (0.6 mm high and 25 mm wide) into a ribbon shape cut via an external device to square pieces of approximately 25 mm × 25 mm in dimensions. We controlled the extrusion process using a control panel enabling adjustment of temperature in selected sections of the extruder, or of the rotational speed of the screw. The snack pellets were then dried in a laboratory dryer at 40 °C to a final moisture content of 8–10%. We stored the dry snack pellets in plastic bags before collection for further tests. If necessary, samples were ground to a particle size of less than 300 µm.

### 3.3. Chemical Composition and Fatty Acids

We tested the proximate composition for dry matter and basic nutrient content in ground samples following the standard procedures of AACC [1995] [59] and AOAC [2011] [60]. The content of protein was assessed according to AACC method 46–10, fat content according to AACC method 30–10, and ash content according to AACC 08–01 method. All work was executed in triplicate. We assessed crude fiber according to the AOAC 993.21 method [60]. Available carbohydrates were calculated by the difference of 100 and the sum of dry matter, proteins, fat, ash and fiber contents.

We determined fatty acids content using gas chromatography (CP WAX 52CB DF 0.25 mm capillary column with 60 m length, chromatograph CP-3800, Varian Medical Systems, Palo Alto, CA, USA) according to AOAC method [1990] [61] after fats conversion to fatty acids methyl esters (FAME). During fatty acids separation, chromatograph operating conditions were helium as a gas carrier, 1.4 mL min^−1^ of flow rate, the temperature of the column at 120 °C increasing gradually 20 °C min^−1^, 127 min time of determination, 160 °C temperature of the feeder, 160 °C temperature of the detector, and oxygen and hydrogen as other gases. We used the Supelco 37-Component Fame Mix template (Sigma-Aldrich, Poznań, Poland) as the basis for the determination procedure. In our work, we assessed the amount of individual fatty acids in the total (100%) value of fatty acids. The saturated and mono- and polyunsaturated fatty acids contents were expressed.

### 3.4. Extraction Procedure

In order to obtain the extracts, we applied ultrasonic extraction (Bandelin Electronic GmbH & Co. KG, Berlin, Germany) with the following parameters: 60 °C, ultrasound frequency of 33 kHz and a power of 320 W). The extracts were prepared from 4 g of milled extrudates which were mixed with 99.8% CH3OH (80 mL) and put in an ultrasonic bath for 40 min. The second stage of the extraction process was based on filtration and repeating the entire extraction process using the same parameters. Afterward, we combined both portions of extracts and evaporated this to dryness. The obtained residue was then dissolved in 10 mL of methanol. The samples were examined according to their total phenolic content, the sum of free phenolic acids and free radical scavenging activity [62].

### 3.5. Phenolic Acids Content

We assessed phenolic acid content using a Waters ACQUITY UPLC Chromatograph (Waters Corp., Milford, MA, USA), equipped with a PDA and a triple-quadrupole mass detector (Waters Corp.). Samples (50 mg mL^−1^) were separated on a Waters ACQUITY UPLC^®^ HSS C18 column (100 mm long × 2.1 mm wide, size of the bed particles 1.8 µm) at 30 °C. The mobile phase consisted of solvent A (0.1% formic acid in water MiliQ) and solvent B (acetonitrile with 0.1% formic acid). Analytes were eluted using the combination of isocratic and gradient steps. We carried out the elution (0.50 mL min^−1^) using a gradient of solvent B: 0–0.5 min, 8% B; 0.5–8 min, 8–20% B; 8–8.10 min, 20–95% B; 8.10–10 min, 95% B; 10–10.10%, 95–8% B; 10.10–12 min, 8% B. The sample injection volume was 2.5 μL (full loop mode). We performed the process in the negative ionization mode, with the use of a selected reaction monitoring method. The condition of MS analysis was published by Czaban et al. [63]. We calculated concentrations of phenolic acids on the basis of calibration curves (Supplementary Materials: Table S1).

We carried out the studies using a modified Folin–Ciocalteu (FC) method. Namely, we first combined 200 μL of extract with 1.8 mL of distilled water. Afterwards, 200 μL of FC reagent was added, mixed and left for 5 min. In the next step, 2 mL of 7% Na_2_CO_3_ was added and the mixture was incubated for 60 min at 40 °C. We recorded absorbance spectrophotometrically using a UV-VIS spectrophotometer Genesys 20 UV-VIS (Thermo Scientific, Waltham, MA, USA) at 760 nm. The obtained results were presented as μg gallic acid equivalents (GAE) per g of dry mass.

### 3.6. Anthocyanins Extraction, Samples Preparation and Quantitative Analysis

We extracted the anthocyanins by following the method of Ramić et al. [64]. The performed ultrasound-assisted extraction was undertaken under the following conditions: 0.1% acidified HCL 50% ethanol solvent (100 mL), 216 W sonication, 70 °C extraction temp. and 45.6 min extraction time. For quantitative analysis of specific anthocyanins by UPLC, we extracted samples by adding 20 mL of formic acid (1:10, *v*/*v* in distilled water) to 4 g of sample (1 g for concentrate samples). After centrifugation at 1000× *g* for 10 min, these extracts were purified on DSC—18 SPE columns (Discovery 52606-U, Sigma Aldrich Chemie, St. Louis, MO, USA). Detained anthocyanins were then leached by washing with 2 mL of methanol in evaporating flasks. Methanol was evaporated in a vacuum evaporator and the residue was dissolved in 1 mL of 0.01% HCl.

We assessed the anthocyanins content in the derived extracts by utilizing reversed-phase ultra-high pressure liquid chromatography, performed on a Waters ACQUITY UPLC^®^ Systems chromatograph (Waters Corporation, Milford, MA, USA) equipped with a photodiode array detector and coupled to a triple-quadrupole mass spectrometer (Waters ACQUITY^®^ TQD, Micromass, Manchester, GB, UK). Samples were then separated on a Waters ACQUITY UPLC^®^ BEH C18 column (1.0 mm × 100 mm; 1.8 μm) at 50 °C. The mobile phase consisted of 0.1% formic acid in MilliQ water (*v*/*v*) and 0.1% formic acid in acetonitrile (*v*/*v*). The analytes were eluted using a linear gradient. We carried out the elution (0.35 mL min^−1^) via a gradient of solvent B: 0.00–0.50 min, 8%; 0.50–8.50 min, 8–60% B; 8.50–8.60 min, 60–90% B; 8.60–9.60 min, 99% B; 9.60–9.70 min, 99–8% B; 9.70–12 min, 8% B. The sample injection volume was 5 μL (full loop mode)

We performed anthocyanins detection in the positive ionization mode, using a selected reaction monitoring method. The source temperature was 120 °C, while the desolvation temperature was 350 °C. We used nitrogen as a desolvation gas (a flow of 1000 L h^−1^) and as a cone gas (100 L h^−1^). Argon was used as a collision gas (0.1 mL min^−1^). The energy of collision was 10 eV. We calculated the concentrations of anthocyanins in chokeberry on the basis of calibration curves (Supplementary Materials: Table S2).

Compound identification was accomplished by comparison of retention time and mass spectral data of the detected anthocyanins with those of authentic standards.

### 3.7. Antioxidant Activity by DPPH Method

In this part of our experiment, we assessed the ability to scavenge free radicals by using the DPPH method (2,2-diphenyl-1-picrylhydrazyl). The method, first presented by Burda and Oleszek [65], was slightly modified (parameters: 517 nm wavelength, calibration based on pure methanol, absorbance recording every 5 min for 30 min). The obtained study results are the average of three measurements. The study results are presented in the form of percentage free radical scavenging as well as TEAC Trolox equivalent antioxidant activity (TEAC). We used the following calibration curve: y = −0.0214x + 0.471. We calculated the free radical scavenging activity by applying the Formula (1):

(1) $$\%=\frac{A_{0}-A_{1}}{A_{0}}\times 100\;[\%]$$

where: A_0_—absorbance of sample (DPPH) without tested extract; A_1_—absorbance of sample (DPPH) with tested extract.

### 3.8. Water Absorption Index (WAI)

We determined WAI using the modified method described by Attenborough et al. [66]. Here, a 10% suspension was prepared from ground snack pellets and distilled water in three replications. The suspension after 10 min stirring was separated by centrifugation at 15,000 rpm for 10 min in the laboratory Digicen 21 centrifuge (Labsystem, Kraków, Poland). After centrifugation, we collected the liquid from the obtained gel and the gel was weighed. Subsequently, we calculated the water absorption index (WAI) using Formula (2):

(2) $$WAI=\frac{m_{z}}{m_{pp}}\;[g\;g^{-1}]$$

where: m_pp_—ground snack pellets mass [g], m_z_—gel mass [g].

### 3.9. Water Solubility Index (WSI)

In this part of the study, we derived WSI in triplicate using the method described by Attenborough et al. [66]. Here, the liquid obtained after the WAI measurement was dried at 130 °C until complete evaporation. We then calculated the water solubility index by using the Formula (3):

(3) $$WSI=\frac{m_{s}-m_{ps}}{m_{pp}}\times 100\;[\%]$$

where: m_s_—vessel mass after drying [g], m_ps_—vessel and liquid mass before drying [g], m_pp_—ground snack pellets mass [g].

### 3.10. Fat Absorption Index (FAI)

We determined the FAI in three replications according to the method described by Lisiecka et al. [48]. We fried samples, i.e., dried snack pellets in vegetable oil at 190 °C until the product completed expansion. The fried product was then drained and weighed to measure the final weight. We tested for moisture content in dry and fried pellets in the meantime and we then assessed the fat absorption index in dry mass using the Formula (4):

(4) $$FAI=\frac{P_{f}-P_{p}}{P_{p}}\times 100\;[\%]$$

where: P_p_—snack pellet dry mass before frying [g], P_f_– snack dry mass after frying and draining oil [g].

### 3.11. Color Profile

We measured the color profile in the CIE-Lab color scale in five replications for each ground sample by using a NR20XE colorimeter (Shenzhen, China), and determined the L*, a* and b* coordinates. Herein, L* values measure lightness (0 black to 100 white); a* values indicate greenness when negative and redness when positive; b* values indicate blueness when negative and yellowness when positive. Additionally, we determined the total color difference index (Δ*E*) using the Formula (5) [67]:

(5) $$\Delta E=\sqrt{{\left(L_{sample}^{*}-L_{control}^{*}\right)}^{2}+{\left(a_{sample}^{*}-a_{control}^{*}\right)}^{2}+{\left(b_{sample}^{*}-b_{control}^{*}\right)}^{2}}$$

### 3.12. Statistical Analysis

We subjected all data to a one-way analysis of variance (ANOVA), followed by the Tukey post hoc test to compare means at the 0.05 significance level by using Statistica 13.3 software (StatSoft, Inc., Tulsa, OK, USA). We PCA and correlation determination at the significance level of 0.05. We used Statistica software (version 13.0, StatSoft Inc., Tulsa, OK, USA) for statistical analyses. The PCA data matrix for the statistical analysis of test results was composed of 21 columns and 12 rows. We determined the number of principal components based on the Cattel criterion, and the input matrix was scaled automatically. For the exploratory data analysis, the aim was to arrange objects into groups in such a way that the degree of linking objects with objects belonging to the same group was as large as possible, and with objects from other groups as small as possible, we applied Ward’s cluster analysis.

## 4. Conclusions

On the basis of the performed study, it can be concluded that fresh chokeberry is a component with great potential to influence the nutritional value of food snacks. Proximate composition, as well as fatty acids profile, showed a positive effect of the application of fresh chokeberry addition in potato-based snack pellets. Along with the addition of 30% of chokeberry to the extrudates, we observed an increase in the content of nutritional components in relation to those containing 10% of chokeberry and to the control sample produced without it, for both 60 and 100 rpm screw speeds. We noted an increase in crude ash- and associated likely increase in micro and macro elements, and in total protein—a desirable dietary component of snacks. Increasing the content of chokeberries as a component used to produce extrudates from 10 to 30% increased the proportion of PUFAs, while reducing the content of SFAs in the total fatty acids, which undoubtedly enhances the nutritional content of this type of snack. Total phenolic content and antioxidant activity were mostly dependent on the amount of additive with no negative effect of processing conditions on the nutritional value of the tested snack pellets. The impact is the most evident for samples enriched with 30% of fruits, however, the increase in antioxidant activity was also significant with the addition of 10% of fruits. TEAC indicated that samples prepared using 60 rpm screw speed and 32% of moisture level reveal the highest antioxidant activity. All the tests carried out confirmed the assumption that the applied production parameters will not adversely affect the antioxidant properties and the content of polyphenols in the tested samples. In this study, we found that the application of fresh chokeberry significantly limited fat absorption and strongly affected the color profile of fortified snack pellets in comparison to control potato-based pellets. Hence, we conclude that the addition of up to 30% fresh chokeberry fruit and the application of appropriate product parameters will generate an extrudate product quite suitable for the functional food market product. In the future, for a more detailed analysis of the optimized food samples, more detailed studies will be carried out using other methods such as ORAC, FRAP or others in snack pellets, but also in ready-to-eat products expanded by way of frying, microwaving or hot-air toasting.

## Figures and Tables

**Table 1:** Basic composition of snack pellets with the addition of fresh chokeberry processed at various conditions.

Additive Content [%]	Moisture Level [%]	Screw Speed [rpm]	Component Content [g 100 g^−1^]					
			DM	CA	CP	CF	CFB	C
0	32	60	89.94 ^d^ ± 0.08	3.76 ^a^ ± 0.03	3.64 ^b^ ± 0.02	0.07 ^a^ ± 0.01	0.31 ^d^ ± 0.05	82.16
		100	89.21 ^a^ ± 0.07	3.76 ^a^ ± 0.04	3.57 ^b^ ± 0.04	0.09 ^ab^ ± 0.02	0.12 ^b^ ± 0.02	81.67
	36	60	89.98 ^d^ ± 0.08	3.76 ^a^ ± 0.06	3.56 ^b^ ± 0.03	0.11 ^b^ ± 0.01	nd	82.55
		100	89.14 ^a^ ± 0.09	3.70 ^a^ ± 0.05	3.46 ^a^ ± 0.03	0.11 ^b^ ± 0.02	0.04 ^a^ ± 0.01	81.84
10	32	60	89.68 ^c^ ± 0.08	4.06 ^c^ ± 0.04	4.13 ^d^ ± 0.04	0.16 ^c^ ± 0.03	nd	81.34
		100	89.50 ^b^ ± 0.08	4.04 ^c^ ± 0.03	4.06 ^c^ ± 0.05	0.10 ^b^ ± 0.02	nd	81.31
	36	60	89.51 ^b^ ± 0.10	4.15 ^d^ ± 0.02	4.05 ^c^ ± 0.02	0.06 ^a^ ± 0.01	nd	81.26
		100	89.36 ^b^ ± 0.08	3.99 ^b^ ± 0.03	4.05 ^c^ ± 0.03	0.16 ^c^ ± 0.03	nd	81.15
30	32	60	90.07 ^e^ ± 0.07	4.88 ^e^ ± 0.06	4.75 ^d^ ± 0.04	0.15 ^c^ ± 0.03	0.09 ^b^ ± 0.01	80.21
		100	89.95 ^d^ ± 0.09	5.07 ^f^ ± 0.05	4.82 ^d^ ± 0.03	0.16 ^c^ ± 0.02	0.17 ^c^ ± 0.02	79.74
	36	60	90.31 ^f^ ± 0.09	4.97 ^e^ ± 0.04	4.84 ^d^ ± 0.04	0.21 ^d^ ± 0.02	0.16 ^bc^ ± 0.03	80.13
		100	89.73 ^c^ ± 0.08	4.92 ^e^ ± 0.03	4.87 ^d^ ± 0.04	0.18 ^cd^ ± 0.03	0.14 ^bc^ ± 0.02	79.63

DM—Dry matter; CA—Crude ash; CP—Crude protein; CF—Ether extract; CFB—Crude fiber; C—Carbohydrates; nd—no data; ^a–f^—means indicated with similar letters in columns do not differ significantly at α = 0.05.

**Table 2:** Fatty acid profiles of snack pellets.

Additive Content [%]	Moisture Level [%]	Screw Speed [rpm]	Fatty Acids [Content in 100%]									
			C 16:0	C 18:0	C 18:1 n-9	C 18:2 n-6	C 18:3 n-3	C 20:0	Sum	Σ SFA	Σ MUFA	Σ PUFA
0	32	60	21.37 ^e^ ± 0.16	8.43 ^i^ ± 0.04	51.22 ^d^ ± 0.15	17.60 ^e^ ± 0.12	0.79 ^d^ ± 0.07	0.00	100	30.38	51.23	18.39
		100	21.94 ^e^ ± 0.12	8.98 ^k^ ± 0.05	51.38 ^d^ ± 0.12	16.55 ^d^ ± 0.16	0.53 ^b^ ± 0.05	0.00	100	31.55	51.38	17.08
	36	60	22.02 ^e^ ± 0.09	8.25 ^h^ ± 0.03	52.69 ^e^ ± 0.11	15.89 ^c^ ± 0.11	0.53 ^b^ ± 0.04	0.00	100	30.88	52.69	16.42
		100	21.20 ^e^ ± 0.11	8.75 ^j^ ± 0.06	54.82 ^g^ ± 0.17	14.01 ^b^ ± 0.13	0.61 ^c^ ± 0.03	0.00	100	30.56	54.82	14.62
10	32	60	17.14 ^d^ ± 0.12	3.15 ^b^ ± 0.02	57.57 ^h^ ± 0.18	19.72 ^f^ ± 0.11	1.20 ^e^ ± 0.02	1.22 ^a^ ± 0.02	100	21.50	57.57	20.92
		100	21.08 ^e^ ± 0.16	7.74 ^g^ ± 0.05	58.55 ^i^ ± 0.21	12.63 ^a^ ± 0.08	nd	nd	100	28.82	58.55	12.63
	36	60	11.84 ^d^ ± 0.08	4.00 ^e^ ± 0.02	59.01 ^i^ ± 0.22	21.97 ^g^ ± 0.14	3.18 ^g^ ± 0.03	nd	100	25.84	59.01	15.16
		100	20.24 ^e^ ± 0.14	8.55 ^i^ ± 0.08	53.25 ^f^ ± 0.18	17.75 ^e^ ± 0.11	0.21 ^a^ ± 0.01	nd	100	28.79	53.25	17.96
30	32	60	11.88 ^b^ ± 0.09	3.28 ^c^ ± 0.04	34.79 ^c^ ± 0.12	45.11 ^h^ ± 0.14	3.70 ^h^ ± 0.03	1.23 ^a^ ± 0.01	100	16.39	34.79	48.81
		100	14.48 ^c^ ± 0.10	3.47 ^d^ ± 0.04	31.26 ^a^ ± 0.12	45.53 ^i^ ± 0.18	5.25 ^j^ ± 0.05	nd	100	17.96	31.26	50.78
	36	60	11.74 ^b^ ± 0.09	4.59 ^f^ ± 0.05	31.88 ^b^ ± 0.13	44.86 ^h^ ± 0.16	4.88 ^i^ ± 0.05	2.05 ^b^ ± 0.02	100	18.38	31.89	49.73
		100	9.29 ^a^ ± 0.07	2.17 ^a^ ± 0.02	35.03 ^c^ ± 0.14	51.66 ^j^ ± 0.20	1.84 ^f^ ± 0.02	nd	100	11.46	35.03	53.50

SFA—saturated fatty acids; MUFA—monounsaturated fatty acids; PUFA—polyunsaturated fatty acids; nd—no data; ^a–k^—means indicated with similar letters in columns do not differ significantly at α = 0.05.

**Table 3:** Nutritional value of chokeberry fortified snack pellets processed at variable conditions (*n* = 3, mean ± SD).

Additive Content [%]	Moisture Level [%]	Screw Speed [rpm]	TPC [μg GAE g^−1^]	TEAC [µg g^−1^ Product]
0	32	60	19.10 ^a^ ± 0.12	73.83 ^a^ ± 5.67
		100	23.10 ^c^ ± 0.06	74.77 ^a^ ± 2.89
	36	60	21.70 ^b^ ± 0.07	75.12 ^a^ ± 4.02
		100	21.80 ^b^ ± 0.05	75.35 ^a^ ± 3.99
10	32	60	64.70 ^d^ ± 0.11	148.95 ^b^ ± 7.23
		100	75.50 ^g^ ± 0.99	160.16 ^b^ ± 7.12
	36	60	71.50 ^f^ ± 1.11	151.17 ^b^ ± 6.94
		100	68.70 ^e^ ± 0.89	151.18 ^b^ ± 8.01
30	32	60	231.20 ^h^ ± 10.12	163.67 ^b^ ± 8.11
		100	230.20 ^h^ ± 7.77	152.80 ^b^ ± 6.87
	36	60	252.20 ^i^ ± 9.71	154.91 ^b^ ± 6.23
		100	223.20 ^h^ ± 5.65	160.16 ^b^ ± 8.32

TPC—total phenolic content; TEAC—Trolox equivalent antioxidant capacity; ^a–i^—means indicated with similar letters in columns do not differ significantly at α = 0.05.

**Table 4:** Anthocyanins content in selected samples of snack pellets (*n* = 3, mean ± SD).

Samples		Cyanidin-3-Galactoside	Cyanidin-3-Glucoside	Cyanidin-3-Arabinoside	Cyanidin-3-Xyloside	Control
Production parameters	Results	Content in µg/g of dry weight				
30%, 36 sm, 60 RPM	Mean	276.52 ^a^	LOD	138.26 ^a^	LOD	ND
	SD	2.12		1.06		
	% RSD	0.77		0.77		
30%, 36 sm, 100 RPM	Mean	284.49 ^b^	LOD	142.24 ^b^	LOD	ND
	SD	4.30		2.15		
	% RSD	1.51		1.51		
30%, 32 sm, 100 RPM	Mean	289.51 ^c^	LOD	144.76 ^c^	LOD	ND
	SD	1.29		0.64		
	% RSD	0.45		0.45		

ND—not detected, LOD—limit of detection; ^a–c^—means indicated with similar letters in columns do not differ significantly at α = 0.05.

**Table 5:** Results of selected physical properties of snack pellets enriched with fresh chokeberry processed at variable conditions (*n* = 3 ± SD).

Additive Content [%]	Screw Speed [rpm]	Moisture Level [%]	WAI [g g ^−1^]	WSI [%]	FAI [%]
0	60	32	2.78 ^b–d^ ± 0.58	4.46 ^a^ ± 0.49	39.01 ^e^ ± 7.33
		36	2.73 ^b^ ± 0.15	6.57 ^b^ ± 0.63	40.20 ^e^ ± 4.14
	100	32	3.04 ^d^ ± 0.24	5.36 ^a^ ± 0.56	22.03 ^d^ ± 6.42
		36	2.88 ^c^ ± 0.07	6.18 ^b^ ± 0.69	26.40 ^d^ ± 2.72
10	60	32	3.03 ^d^ ± 0.55	7.27 ^bc^ ± 0.18	22.17 ^d^ ± 2.23
		36	2.51 ^a^ ± 0.11	10.37 ^e^ ± 0.53	20.11 ^d^ ± 3.40
	100	32	2.72 ^b^ ± 0.01	8.15 ^c^ ± 0.23	17.84 ^d^ ± 2.24
		36	2.59 ^a^ ± 0.04	9.25 ^d^ ± 0.31	19.73 ^d^ ± 3.17
30	60	32	2.94 ^c^ ± 0.11	11.56 ^ef^ ± 0.72	3.23 ^a^ ± 0.66
		36	2.75 ^b^ ± 0.07	12.72 ^fg^ ± 0.79	7.68 ^c^ ± 1.56
	100	32	2.93 ^c^ ± 0.04	9.92 ^e^ ± 0.58	5.73 ^b^ ± 1.34
		36	2.70 ^b^ ± 0.12	11.27 ^f^ ± 0.33	5.03 ^b^ ± 1.03

WAI—Water Absorption Index; WSI—Water Solubility Index; FAI—Fat Absorption Index; ^a–g^—means indicated with similar letters in columns do not differ significantly at α = 0.05.

**Table 6:** Results of color coordinated and total color change index of snack pellets enriched with fresh chokeberry (*n* = 5 ± SD).

Additive Content [%]	Screw Speed [rpm]	Moisture Level [%]	L*	a*	b*	Δ*E*
0	60	32	57.94 ^d^ ± 2.41	2.67 ^a^ ± 0.49	12.18 ^e^ ± 0.28	ref
		36	60.77 ^e^ ± 0.75	2.39 ^a^ ± 0.07	11.46 ^d^ ± 0.08	ref
	100	32	56.64 ^d^ ± 0.47	3.10 ^ab^ ± 0.20	13.42 ^f^ ± 0.35	ref
		36	55.44 ^d^ ± 2.75	2.73 ^a^ ± 0.44	10.81 ^d^ ± 0.54	ref
10	60	32	33.67 ^b^ ± 0.31	6.77 ^c^ ± 0.21	1.26 ^b^ ± 0.23	26.94 ^a^ ± 2.41
		36	35.87 ^c^ ± 0.61	8.62 ^d^ ± 1.07	1.98 ^b^ ± 0.30	27.38 ^a^ ± 0.64
	100	32	33.41 ^b^ ± 0.55	6.85 ^c^ ± 1.15	1.79 ^b^ ± 0.39	24.26 ^a^ ± 2.56
		36	35.55 ^c^ ± 0.37	9.48 ^d^ ± 0.08	2.29 ^c^ ± 0.04	24.69 ^a^ ± 0.48
30	60	32	29.25 ^a^ ± 0.46	3.30 ^b^ ± 0.68	0.65 ^a^ ± 0.13	30.94 ^b^ ± 2.51
		36	29.59 ^a^ ± 0.67	3.64 ^b^ ± 0.26	0.84 ^a^ ± 0.05	32.96 ^b^ ± 0.95
	100	32	29.14 ^a^ ± 0.93	2.58 ^a^ ± 0.22	0.64 ^a^ ± 0.11	30.33 ^b^ ± 0.85
		36	29.69 ^a^ ± 0.81	2.97 ^ab^ ± 0.41	0.78 ^a^ ± 0.07	27.69 ^a^ ± 2.33

L*—lightness; a*—redness-greenness balance; b*—yellowness-blueness balance; Δ*E*—total color change index; ^a–f^—means indicated with similar letters in columns do not differ significantly at α = 0.05. Of note: Taskin [51] reported the L*, a* and b* values for fresh black chokeberry as being 15.92, 2.41 and 0.75, respectively.

## Data Availability

Data are available in Department of Food Process Engineering, University of Life Sciences in Lublin, Poland.

## Supplementary Materials

The following supporting information can be downloaded at: https://www.mdpi.com/article/10.3390/plants12183276/s1, Figure S1: Chromatogram of the tested phenolic acids in snack pellets processed at 36 mc and 60 rpm supplemented with 30% of chokeberry; Table S1: Parameters of the calibration curve for nine different phenolic acids; Table S2: Parameters of the calibration curve for nine different anthocyanins.
