# Supplementary material for: Fresh Chokeberry (Aronia melanocarpa) Fruits as Valuable Additive in Extruded Snack Pellets: Selected Nutritional and Physiochemical Properties
Source: Plants (Basel). 2023 Sep 15;12(18):3276. doi: 10.3390/plants12183276 (PMC10537414; doi:10.3390/plants12183276)
Supplement: Supplementary file 1 [file plants-12-03276-s001.zip › plants-2566032-SI.pdf]

plants

Table S1. Parameters of the calibration curve for nine different phenolic acids.

No.	Compound	Calibration Curve	R^2
1	protocatechuic acid	y = – 0.0254 x^2 + 1.4661 x + 0.0137	0.997
2	p–OH–benzoic acid	y = – 0.0117 x^2 + 1.4390 x + 0.1649	0.997
3	vanillic acid	y = 0.0001 x^2 +0.1940 x – 0.0031	0.998
4	caffeic acid	y = – 0.0183 x^2 + 2.4210 x + 0.4368	0.995
5	syryngic acid	y = – 0.00005 x^2 +0.2598 x – 0.0026	0.986
6	p–coumaric acid	y = – 0.0166 x^2 + 2.0582 x + 2.0582	0.993
7	ferulic acid	y = – 0.0004 x^2 + 0.3801 x + 3.3001	0.994
8	synapic acid	y = – 0.0032 x^2 + 0.5524 x – 0.0620	0.998
9	salicylic acid	y = – 0.0338 x^2 + 3.2638 x + 0.8268	0.998

Table S2. Parameters of the calibration curve for nine different anthocyanins.

No.	Compound	Calibration Curve	R2	Standard
1	cyanidin-3-galactoside	y = 0.0356116 x2 + 1.46712 x + 0.028605	0.998	Sigma Aldrich
2	cyanidin-3-glucoside	y = – 0.0234753 x2 + 1.67904 x + 0.133956	0.998	Sigma Aldrich
3	cyanidin-3-arabinoside	y = 0.02451638 x2 + 0.6744029 x – 0.05311	0.998	Sigma Aldrich
4	cyanidin-3-xyloside	y = 0.04351638 x2 + 0.6327 x – 0.43343	0.998	Sigma Aldrich

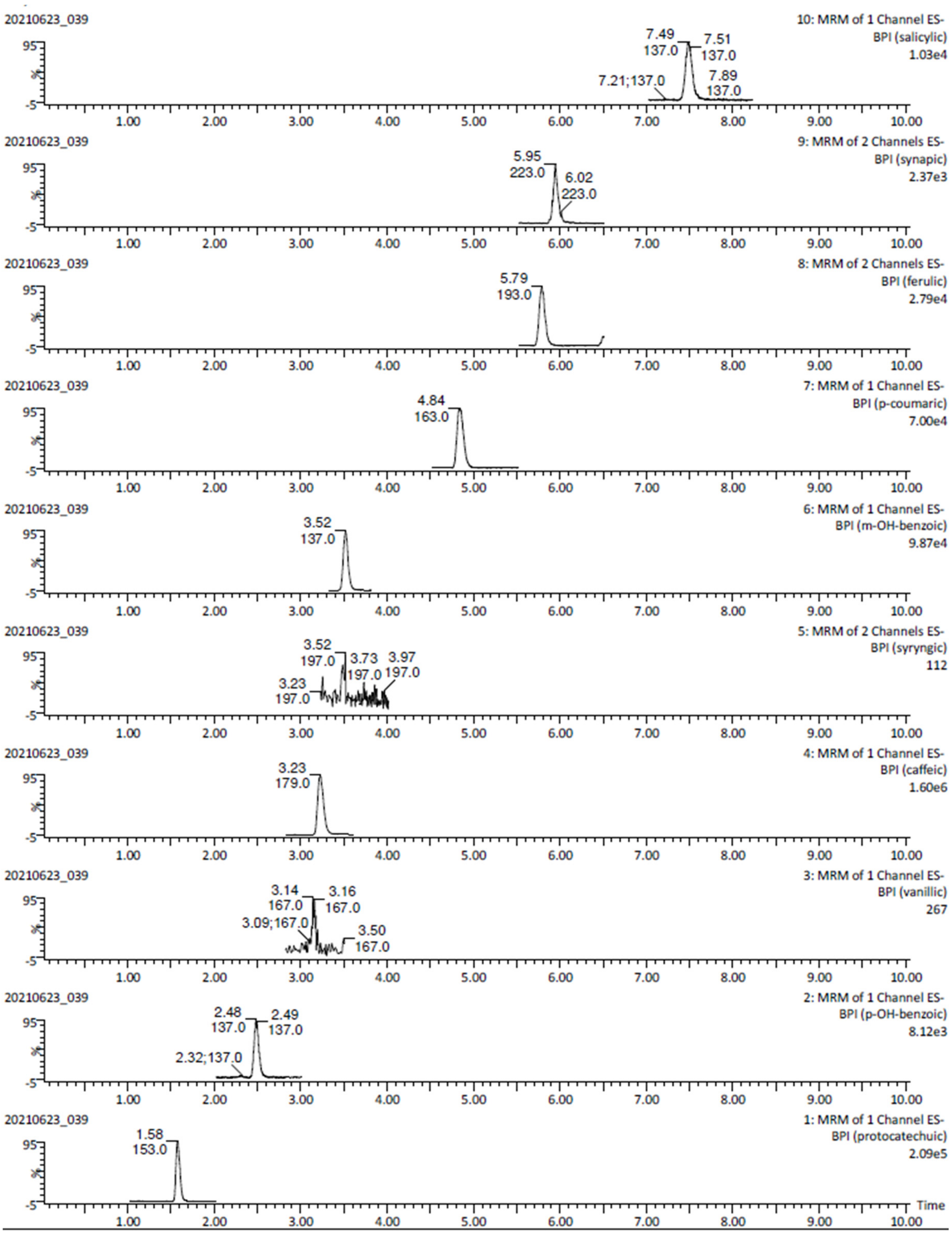

Figure S1. Chromatogram of the tested phenolic acids in snack pellets processed at 36 mc and 60 rpm supplemented with 30% of chokeberry.
